# Supplementary material for: Determination of Multiclass Cyanotoxins in Blue-Green Algae (BGA) Dietary Supplements Using Hydrophilic Interaction Liquid Chromatography-Tandem Mass Spectrometry
Source: Toxins (Basel). 2023 Feb 4;15(2):127. doi: 10.3390/toxins15020127 (PMC9960112; doi:10.3390/toxins15020127)
Supplement: Supplementary file 1 [file toxins-15-00127-s001.zip › toxins-2121084-supplementary.pdf]

toxins

MDPI

Supplementary Materials: Determination of Multiclass Cyanotoxins in Blue-Green Algae (BGA) Dietary Supplements Using Hydrophilic Interaction Liquid Chromatography-Tandem Mass Spectrometry

María del Mar Aparicio-Muriana, Francisco J. Lara, Monsalud Del Olmo-Iruela and Ana M. García-Campaña

Table S1. List of analyzed BGA dietary supplements.

Sample	Supplier	Form	Composition	Daily dose
1	Local store	Powder	Pure spirulina (A*throspira Platensis*)	6000 mg spirulina
2	Online	Capsule	Spirulina (*Platensis Gomont*), Hydroxypropyl methylcellulose, chlorella (*chlorella vulgaris Beijerinck*), fucus (*fucus vesicculosus L.*), vitamin C, magnesium stearate	891 mg spirulina, 445.5 mg chlorella, 445.5 mg fucus
3	Online	Tablet	Spirulina (*Arthrospira platensis*), Chlorella (*chlorella vulgaris*), ferrous fumarate, folic acid, vitamin B12	750 mg spirulina, 750 mg chlorella
4	Online	Tablet	Klamath (*Aphanizomenon flos-aquae*), spirulina (*Spirulina Platensis Geitler*), microcrystalline cellulose, silicon dioxide	600 mg klamath, 600 mg spirulina
5	Online	Tablet	Chlorella (C*hlorella Pyrenoidosa Chick*), silicon dioxide	3268 mg chlorella
6	Online	Tablet	Spirulina (*Spirulina Platensis Geitler*), dioxide de silício	2562 mg spirulina
7	Local Store	Capsule	Spirulina (*Arthrospira Platensis*), hypromellose	2160 mg spirulina
8	Local Store	Powder	Pure spirulina	Not available
9	Local Store	Powder	Pure klamath	Not available

Toxins **2023**, *15*, 127. https://doi.org/10.3390/toxins15020127

Table S2. Toxicity, structure and physico-chemical properties of target cyanotoxins

Toxin group	Toxicity	Cyanotoxin	pKa[a]	pKa[a]	log P[a]	Chemical Structure
Cyclic peptides	Hepato toxin	Microcystin-leucine-arginine (MC-LR)	2.2	12.4	2.4	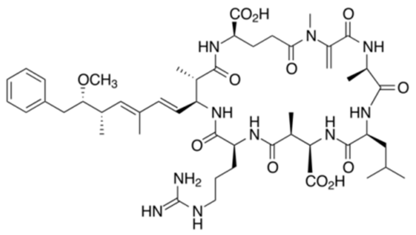
		Microcystin-arginine-arginine (MC-RR)	3.0	13.7	−0.22	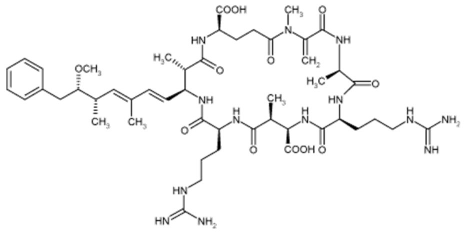
		Nodularin (NOD)	3.4	10.8	1.5	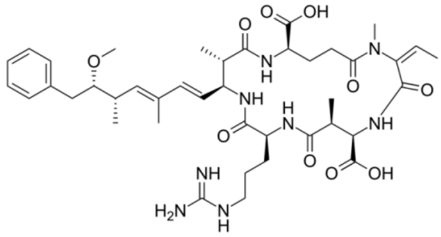
Alkaloids	Neurotoxin	Anatoxin-a (ANA)		9.6	0.8	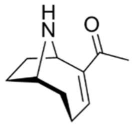
Non-protein amino acids	Neurotoxin	β-methylamino-L-alanine (BMAA)	2.1	6.6	−0.1	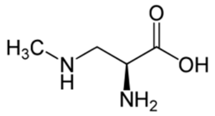
		2,4-diaminobutyric acid (DAB)	2.6	8.4	−4.0	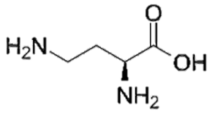
		N-(2-aminoethyl)glycine (AEG)	2.2	9.8	−3.8	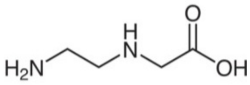

[a] Advanced Chemistry Development software V11.02 (ACD/Labs, Toronto, Ontario, Canada).

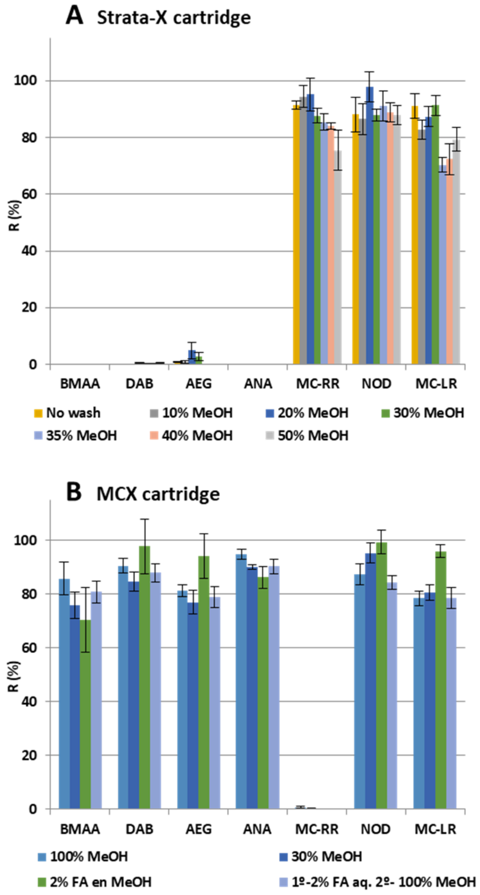

Figure S1. Evaluation of washing solvents in the tandem-SPE procedure. (**A**) Strata-X cartridge; (**B**) MCX cartridge.

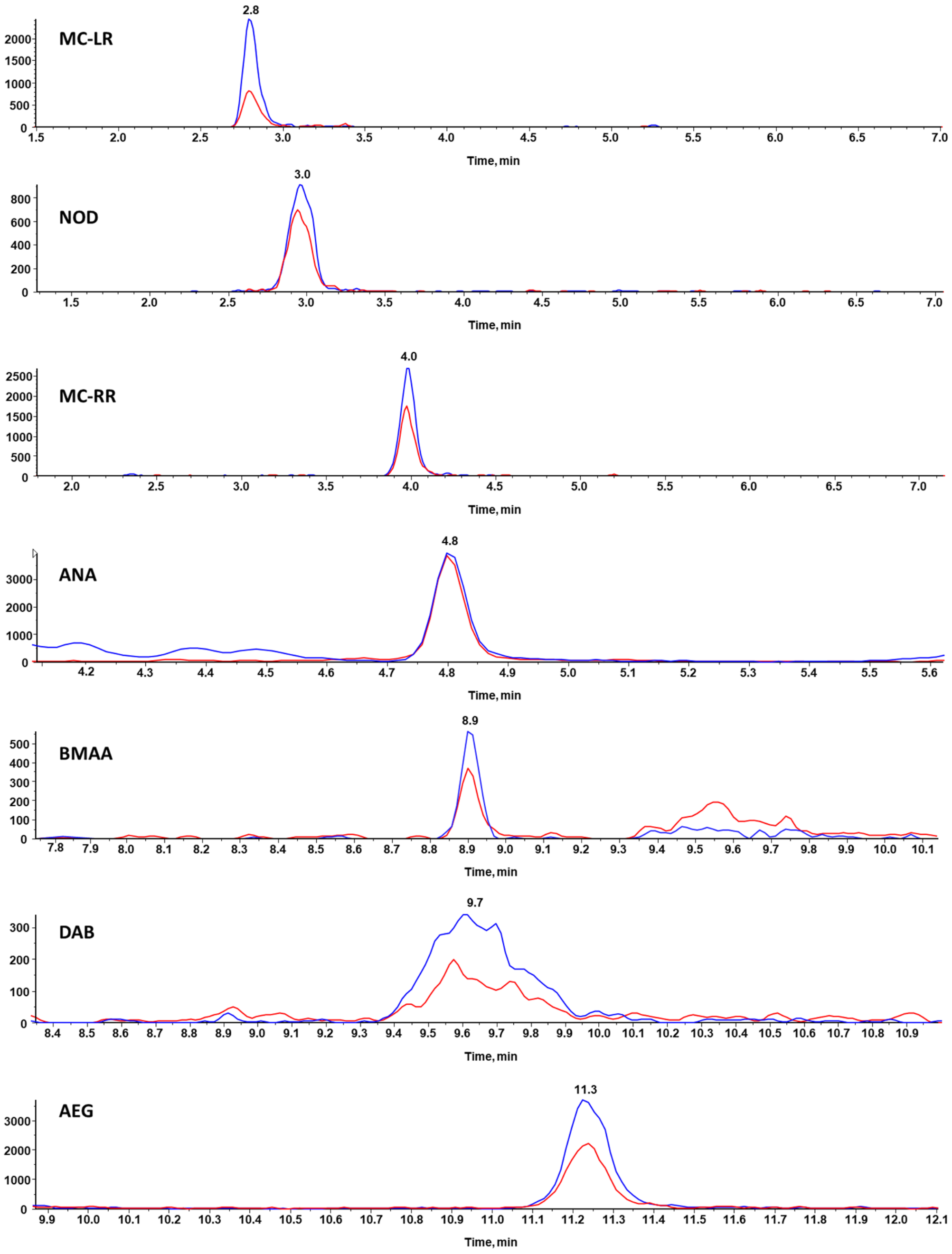

Figure S2. Chromatographic separation of a blank BGA dietary supplement sample spiked with a mixture of cyanotoxins at a concentrations corresponding with the fourth point of the calibration curve. Blue and red lines represent the Q_{ion} and I_{ion} transitions, respectively.
